# Association Between Serum Vitamin A Levels and Recurrent Respiratory Tract Infections in Children

**DOI:** 10.3389/fped.2021.756217

**Published:** 2021-12-24

**Authors:** Xiaoyan Wang, Xingming Li, Chunhua Jin, Xinyuan Bai, Xinran Qi, Jianhong Wang, Lili Zhang, Na Li, Na Jin, Wenhong Song, Haitao Gao, Baojun Gao, Yue Zhang, Lin Wang

**Affiliations:** ^1^Department of Children Health Care, Capital Institute of Pediatrics, Beijing, China; ^2^School of Public Health, Capital Medical University, Beijing, China

**Keywords:** vitamin A, RRTIs, BMI, children, adolescents, Chinese

## Abstract

To evaluate the association between serum vitamin A levels and the prevalence of recurrent respiratory tract infections (RRTIs) in children and adolescents and to provide evidence that would help decrease the prevalence of respiratory tract infections (RTIs) in children. This cross-sectional study included 8034 children and adolescents in Beijing aged 6 months to 17 years. RRTI and RTI symptoms were diagnosed according to the *Clinical Concept and Management of Recurrent Respiratory Tract Infections in Children*. Multivariate logistic regression models were used to evaluate the association between serum vitamin A levels and RRTIs after adjusting for potential confounders. Among the included children, 721 (8.97%) were diagnosed with vitamin A deficiency, whereas 3,073 (38.25%) were diagnosed with subclinical vitamin A deficiency. Only 28.8% (208/721) of children with vitamin A deficiency and 53.1% (1,631/3,073) of children with subclinical vitamin A deficiency had no RRTI and RTI symptoms, respectively. Compared with children with normal vitamin A levels, those with vitamin A deficiency and subclinical vitamin A deficiency had a greater risk for RRTIs, with an odds ratio (OR) of 6.924 [95% confidence interval (CI): 5.433–8.824] and 2.140 (95% CI: 1.825–2.510), respectively]. Vitamin A levels were also positively associated with RTI symptoms, with those having vitamin A deficiency and subclinical vitamin A deficiency showing an OR of 1.126 (95% CI: 0.773–1.640) and 1.216 (95% CI: 1.036–1.427), respectively. The present cross-sectional study found that low serum vitamin A levels were significantly associated with RRTI or RTI prevalence in children and adolescents.

## Introduction

Respiratory tract infections (RTIs) are the most widespread infectious disease in children and adolescents and a serious public health concern promoting high morbidity and mortality that considerably threatens children's health. Preschool-aged children documented to have suffered more than eight episodes of airway infections per year or older children who have suffered from more than six respiratory infections are considered to have recurrent respiratory tract infections (RRTIs) (1). Furthermore, the average frequencies of infectious episodes in children with RRTIs aged 0–2, 3–5, and 6–14 years are more than 7, 6, and 5 times a year, respectively (2). In 2008, the Editorial Committee of the Chinese Journal of Pediatrics and the Department of Respiratory in the Chinese Pediatric Society of Chinese Medical Association established the official criteria for diagnosing RRTIs (3).

Besides the traditional risk factors of RRTIs, including parental educational level, family medical history of respiratory diseases, parental smoking status, nutritional status, supplemental microelements, immune status, diet status, exercise, and environmental factors (4), previous evidence has suggested that low serum vitamin A levels are significantly associated with high morbidity of RRTIs and respiratory infections (5–7). Consistent with these findings, it has been found that patients with chronic infectious diseases generally present with severe vitamin A deficiency (8). Additionally, a clinical trial on 100 children with RRTI without acute phase response demonstrated that vitamin A supplementation could decrease the morbidity of RRTIs (9).

At present, most of the research on this topic has focused on preventing the occurrence or recurrence of RRTIs, especially in preschool children. However, current evidence suggests that vitamin A supplementation has only a limited effect in preventing acute respiratory infections and may not be suitable for all children (10). Therefore, a well-designed observational study of children and adolescents that comprehensively evaluates serum vitamin A levels and RRTIs is warranted before utilizing vitamin A supplements for the prevention of RRTIs in children. Therefore, we conducted a cross-sectional study to explore the association between serum vitamin A levels and the prevalence of RRTIs in children and adolescents.

## Materials and Methods

This cross-sectional study included eight pediatric healthcare centers in Beijing, China, namely the Capital Institute of Pediatrics, Beijing Shouer Liqiao Children's Hospital, Beijing Fangshan District Maternal and Child Health Hospital, Civil Aviation General Hospital, Beijing Shijingshan District Maternal and Child Health Care Hospital, Beijing Pinggu District Maternal and Child Health Care Hospital, Tsinghua University Yuquan Hospital, and Beijing Huairou Hospital. All children (aged <18 years old) who visited the pediatrician's office for the treatment of rhinitis, influenza, pharyngitis, amygdalitis, trachitis, bronchitis, pneumonia, or other respiratory tract diseases or for a routine physical examination between April 2012 and May 2017 were included in this study. The study protocol was approved by the Ethics Committee of the Capital Institute of Pediatrics. Participants and participants' families were informed before the investigation, and children's parents provided written informed consent for research enrollment and data accessibility.

Patients with acute or chronic inflammation of other systems or organs, congenital diseases, abnormal immune system diseases, or a history of surgery were excluded from this study.

RRTI and RTI symptoms were diagnosed according to the criteria detailed in *Clinical Concept and Management of Recurrent Respiratory Tract Infections in Children* edited by the Chinese Pediatric Society of Chinese Medical Association in 2008 (2) and relative information extracted from the medical records of the participants. Two professional physicians reconfirmed the patient information and finally diagnosed RRTI and RTI symptoms. Children and adolescents aged between 6 months and 17 years who had respiratory infections or visited the pediatrics or child health department of the abovementioned hospitals for health examinations constituted the study population. Patients with infections of other systems (except the respiratory tract), congenital diseases, autoimmune diseases, and a history of surgery were excluded. Those children whose parents/guardians did not provide consent for participation in the study were also excluded. Participants were divided into the following four groups: healthy children without a history of RRTIs or any RTI symptoms, patients with RRTI presenting with RTI symptoms, patients with RRTI without RTI symptoms, and patients without RRTI presenting with respiratory disease symptoms.

For each participant, 3 mL of peripheral venous blood was collected into thrombin blood collection tubes (orange top) after overnight fasting. Blood collection was performed by professional nurses. Collected blood samples were centrifuged at 3,000 rpm/min for 10 min and left to settle down. The supernatant was transferred into a clean microcentrifuge tube. Collected serum was stored at −20°C until analysis. Serum vitamin A concentrations were assessed by Beijing Harmony Health Medical Diagnostics Co., Ltd. using HPLC (LC-20A; Shimadzu, Japan). Vitamin A concentrations of >0.3, <0.3, and <0.2 mg/L were defined as normal, subclinical deficiency, and deficiency, respectively, according to the deficiency levels defined by the World Health Organization (11).

The demographic information and characteristics of the participants, as well as information regarding residential conditions and medical histories, were reported by the participants' parents through a predesigned questionnaire. All included children underwent standard physical examination, including height, weight, and temperature measurements and routine blood examinations. The body mass index (BMI) was calculated as body weight (kg)/height squared (m^2^). According to the criteria defined by World Health Organization (12), children were categorized as normal, under weight, overweight, or obesity. All procedures performed herein, including the questionnaire interview, physical examination, and RRTI and RTI symptom diagnosis, were performed by professional pediatricians.

### Statistical Analysis

Categorical variables were presented as frequencies and proportions. Chi-squared test was used to compare differences between the characteristics of children with vitamin A deficiency, subclinical deficiency, and no deficiency. Multivariate logistic regression models were created to evaluate the association between serum vitamin A levels and respiratory diseases (RRTI/RTI group vs. healthy group, combined RTI and non-RTI group vs. healthy group, non-RRTI and RTI group vs. healthy group, combined RRTI and RTI group vs. healthy group) after adjusting for potential confounders, including age, sex, residential location, BMI, and food types (most consumed foods: staple food, vegetables, eggs white or egg yolks, red meat, and fruits). After stratifying the children according to sex, age, residential location, BMI, and food types, a subgroup analysis was performed to identify populations with potentially increased benefits. The odds ratios (ORs) and their 95% confidence intervals (CIs) were calculated.

All data analyses were conducted using statistical software SPSS version 23.0 (SPSS, Chicago, IL, USA), with two-tailed *P* < 0.05 indicating statistical significance.

## Results

**Figure 1:**
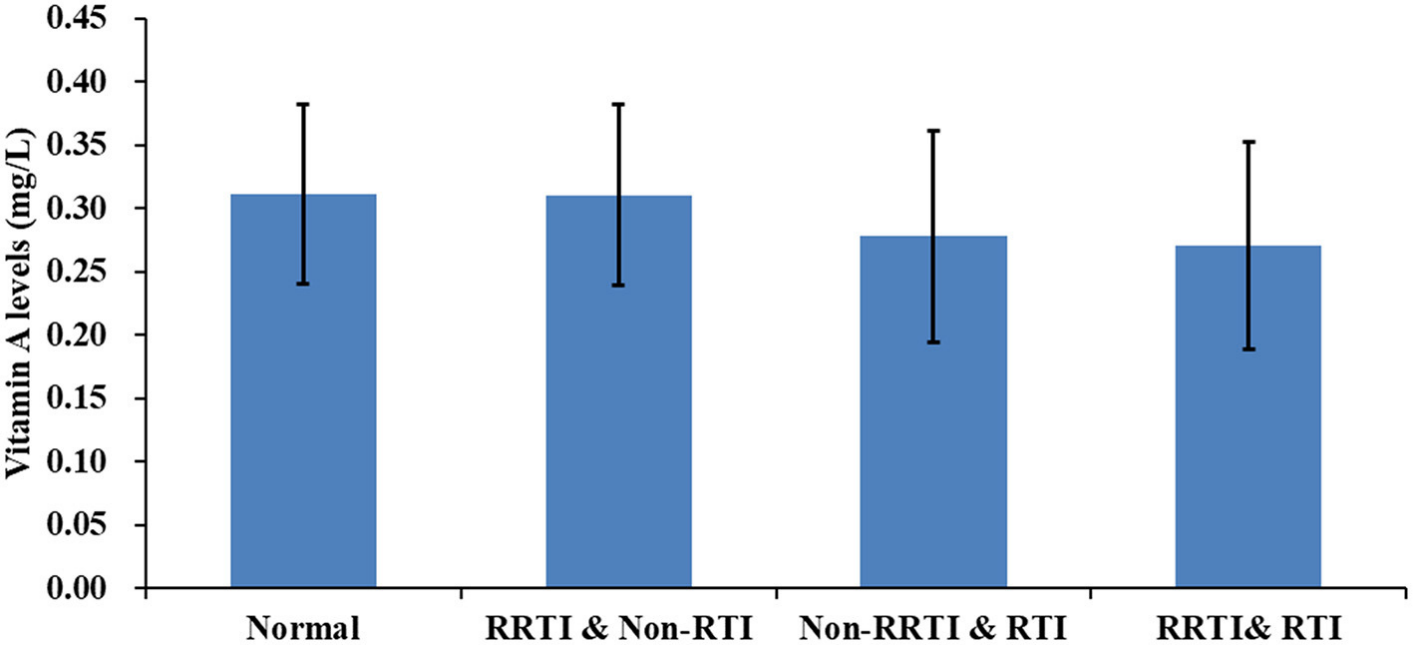
Histogram of vitamin A levels in children with recurrent respiratory infections and normal children.

Among the 8,034 children and adolescents aged between 6 months and 17 years, 58.8% were boys, 95.8% were of Han ethnicity, and 85.7% resided in urban areas (Table 1). Only 5.4% of the children were underweight, whereas 83.5% had a healthy weight. Further, 721 (8.97%), 3,073 (38.2%), and 4,240 (52.8%) children had vitamin A deficiency, subclinical vitamin A deficiency, and healthy serum vitamin A levels, respectively. In addition, 10.6, 21.4, and 12.3% of the children were diagnosed with RRTI, RTI symptoms, and RRTI with RTI symptoms, respectively. Compared with children with healthy vitamin A levels, children with vitamin A deficiency and subclinical vitamin A deficiency had RRTI or RTI symptoms more frequently (71.2 and 46.9%, respectively; *P* < 0.001). Serum vitamin A levels were 0.31 mg/L (±0.07), 0.28 mg/L (±0.08), and 0.27 mg/L (±0.08) in children with only RRTI, only RTI symptoms, and both RRTI and RTI symptoms, respectively (Figure 1).

**Table 1:** Characteristics of 8,034 children and adolescents according to the serum vitamin A levels.

**Characteristics**	**Total**	**Vitamin A levels**			***P***
		**Deficiencies <0.2 mg/L**	**Subclinical deficiencies 0.2 **~ < ** 0.3 mg/L**	**Normal ≥ 0.3 mg/L**	
	*n* = 8,034	*n* = 721	*n* = 3,073	*n* = 4,240	
Boys, *n* (%)	4,726 (58.8)	425 (58.9)	1,825 (59.4)	2,476 (58.4)	0.695
Age, *n* (%)					
6 months ~ <1 year	864 (10.8)	114 (15.8)	427 (13.9)	323 (7.6)	<0.001
1–3 years	2,764 (34.4)	229 (31.8)	1,008 (32.8)	1,527 (36.0)	
3–6 years	2,881 (35.9)	266 (36.9)	1,075 (35.0)	1,540 (36.3)	
6–17.38 years	1,525 (19.0)	112 (15.5)	563,18.3)	850 (20)	
Han ethnicity, *n* (%)	7,695 (95.8)	704 (97.6)	2,929 (95.3)	4,062 (95.8)	0.020
Residential location, *n* (%)					
Migrant population	623 (7.8)	54 (7.5)	249,8.1)	320 (7.5)	<0.001
Rural	526 (6.5)	88 (12.2)	206 (6.7)	232 (5.5)	
Urban	6,885 (85.7)	579 (80.3)	2,618 (85.2)	3,688 (87.0)	
BMI, *n* (%)					
Normal	6,493 (83.5)	551 (77.2)	2,485 (83.6)	3,457 (84.5)	<0.001
Under weight	418 (5.4)	49 (6.9)	173,5.8)	196 (4.8)	
Overweight/obesity	868 (11.2)	114 (16.0)	314 (10.6)	440 (10.8)	
Respiratory diseases, *n* (%)					
Upper respiratory tract infections	6,767 (66.7)	641 (55.6)	2,581 (65.4)	3,545 (70.2)	0.001
Tracheitis	2,173 (21.4)	305 (26.5)	865 (21.9)	1,003 (19.8)	<0.001
Pneumonia	1,210 (11.9)	206 (17.9)	499 (12.6)	505 (10.0)	<0.001
Respiratory conditions, *n* (%)					
Non-RRTI and non-RTI	4,478 (55.7)	208 (28.8)	1,631 (53.1)	2,639 (62.2)	<0.001
RRTI and non-RTI	849 (10.6)	36 (5.0)	328 (10.7)	485 (11.4)	
Non-RRTI and RTI	1,720 (21.4)	298 (41.3)	669 (21.8)	753 (17.8)	
RRTI and RTI	987 (12.3)	179 (24.8)	445 (14.5)	363 (8.6)	
Food consumed types, *n* (%)					
Staple food	5,754 (71.9)	549 (76.8)	2,222 (72.7)	2,983 (70.5)	0.001
Vegetables	315 (3.9)	17 (2.4)	110 (3.6)	188 (4.4)	
Eggs	623 (7.8)	53 (7.4)	250 (8.2)	320 (7.6)	
Meat	611 (7.6)	38 (5.3)	210 (6.9)	363 (8.6)	
Fruits	697 (8.7)	58 (8.1)	264 (8.6)	375 (8.9)	

*RRTI, Recurrent Respiratory Tract Infections; RTI, Respiratory Tract Infections*.

Compared with children with healthy serum vitamin A levels, those with vitamin A deficiency and subclinical vitamin A deficiency were at a greater risk for RRTIs/RTIs [OR for RRTIs/RTI of 4.262 [95% CI: 3.561–5.100; *P* < 0.001) and 1.513 (95% CI: 1.371–1.669; *P* < 0.001), respectively] after adjusting for potential confounders (Table 2). Moreover, compared with children with healthy vitamin A levels, those with vitamin A deficiency and subclinical vitamin A deficiency were at a greater risk for RRTIs [OR for RRTI of 1.188 (95% CI: 0.814–1.733; *P* = 0.372) and 1.193 (95% CI: 1.017–1.399; *P* = 0.030), respectively]. Consistently, children with vitamin A deficiency and subclinical vitamin A deficiency had an OR for RTI symptoms of 5.088 (95% CI: 4.159–6.225; *P* < 0.001) and 1.483 (95% CI: 1.309–1.681; *P* < 0.001), respectively. Our results showed that children with vitamin A deficiency and subclinical vitamin A deficiency had a 7.1 (95% CI: 5.566–9.057; *P* < 0.001) and 2.133 (95% CI: 1.818–2.503; *P* < 0.001) times higher risk for combined RRTI and RTI, respectively.

**Table 2:** Associations between serum vitamin A levels and risk of recurrent respiratory infections in children aged 0.5–17 years in Beijing.

**Serum vitamin A levels**	**Cases/*N* (%)**	**Crude model**	Adjusted model^*^	***P***
RRTI/RTI vs. Healthy children				
Vitamin A levels				
Deficiency	513/721 (71.2)	4.065 (3.421,4.832)	4.262 (3.561,5.100)	<0.001
Subclinical deficiencies	14,423,073/(46.9)	1.457 (1.326,1.601)	1.513 (1.371,1.669)	<0.001
Normal	1,601/4,240 (37.8)	Referent	–	–
Combined RRTI and non-RTI vs. Healthy				
Vitamin A levels				
Deficiency	36/244 (14.8)	0.942 (0.653,1.359)	1.188 (0.814,1.733)	0.372
Subclinical deficiencies	328/1,959 (16.7)	1.094 (0.939,1.275)	1.193 (1.017,1.399)	0.030
Normal	485/3,124 (15.5)	Referent	–	–
Combined Non-RRTI and RTI vs. Healthy				
Vitamin A levels				
Deficiency	298/506 (58.9)	5.021 (4.133,6.100)	5.088 (4.159,6.225)	<0.001
Subclinical deficiencies	669/2,300 (29.1)	1.438 (1.274,1.623)	1.483 (1.309,1.681)	<0.001
Normal	753/3,392 (22.2)	Referent	–	–
Combined RRTI and RTI vs. Healthy				
Vitamin A levels				
Deficiency	179/387 (46.3)	6.256 (4.981,7.858)	7.100 (5.566,9.057)	<0.001
Subclinical deficiencies	445/2,076 (21.4)	1.984 (1.704,2.309)	2.133 (1.818,2.503)	<0.001
Normal	363/3,002 (12.1)	Referent	–	–

* *Adjusted model: adjusted for age, sex, residential location, BMI, and food types*.

After stratifying the patients according to sex, the subgroup analysis showed a consistently negative association between vitamin A levels and risk for RRTIs in both girls and boys (Table 3). After stratifying the patients according to age groups, the analysis revealed an increasing risk for RRTIs among those with vitamin A deficiency as age increased, with children aged <1, 1–3, 3–6, and >6 years showing an OR for RRTIs of 1.498 (95% CI: 0.924–2.429; *P* = 0.101), 5.434 (95% CI: 3.962–7.455; *P* < 0.001), 4.647 (95% CI: 3.316–6.513; *P* < 0.001), and 6.990 (95% CI: 4.329–11.286; *P* < 0.001), respectively. This suggests that the risk for RRTIs in children with subclinical vitamin A deficiency was consistent as age increased. After stratifying the patients according to location, the analysis revealed that the association between vitamin A levels and RRTI risk was stronger among rural and migrant populations, with the ORs being 4.165 (95% CI: 3.415–5.079; *P* < 0.001) and 1.445 (95% CI: 1.297–1.609; *P* < 0.001) for urban children with vitamin A deficiency and subclinical vitamin A deficiency, 7.407 (95% CI: 3.583–15.315; *P* < 0.001) and 2.845 (95% CI: 1.970–4.108; *P* < 0.001) for migrant children with vitamin A deficiency and subclinical vitamin A deficiency, and 6.707 (95% CI: 3.258–13.806; *P* < 0.001) and 2.345 (95% CI: 1.535–3.584; *P* < 0.001) for rural children with vitamin A deficiency and subclinical vitamin A deficiency, respectively. After stratifying the patients according to BMI, the analysis showed a stronger association between vitamin A deficiency and risk for RRTIs among children who were overweight/obese, with the ORs being 7.529 (95% CI: 4.253–13.330; *P* < 0.001) and 2.514 (95% CI: 1.830–3.452; *P* < 0.001) for children who were overweight/obese and had vitamin A deficiency and subclinical vitamin A deficiency, 4.345 (95% CI: 3.548–5.322; *P* < 0.001) and 1.512 (95% CI: 1.356–1.686; *P* < 0.001) for children with healthy weight with vitamin A deficiency and subclinical vitamin A deficiency, 3.204 (95% CI: 1.546–6.638; *P* = 0.002) and 1.028 (95% CI: 0.673–1.570; *P* = 0.897) for children who were underweight with vitamin A deficiency and subclinical vitamin A deficiency, respectively. The association between vitamin A deficiency and RRTIs was consistent in children consuming primarily staple food, vegetables, eggs white or egg yolks, and fruits. However, the children consuming primarily meat products showed a stronger association between vitamin A levels and risk for RRTIs, with an OR of 6.375 (95% CI: 2.545–15.973; *P* < 0.001) and 1.304 (95% CI: 0.909–1.870; *P* = 0.149) for vitamin A deficiency and subclinical vitamin A deficiency, respectively.

**Table 3:** Associations between serum vitamin A levels and risk of respiratory infections in subgroups.

**Serum vitamin A levels *N***	**Cases/ (%)**	**Crude model**	Adjusted model^*^	***P***
Sex				
Boys				
Vitamin A levels				
Deficiency	305/425 (71.8)	3.893 (3.105, 4.881)	3.843 (3.043, 4.854)	<0.001
Subclinical deficiencies	874/1,825 (47.9)	1.408 (1.246, 1.591)	1.408 (1.241, 1.598)	<0.001
Normal	978/2,476 (39.5)	Referent	–	–
Girls				
Vitamin A levels				
Deficiency	208/296 (70.3)	3.303 (2.489, 4.382)	3.389 (2.537, 4.525)	<0.001
Subclinical deficiencies	568/1,248 (45.5)	1.652 (1.347, 2.027)	1.676 (1.360, 2.066)	<0.001
Normal	623/1,764 (35.3)	Referent	–	–
Age				
<1 yrs				
Vitamin A levels				
Deficiency	41/114 (36.0)	1.573 (0.997, 2.480)	1.498 (0.924, 2.429)	0.101
Subclinical deficiencies	104/427 (24.4)	0.902 (0.647, 1.256)	0.829 (0.584, 1.177)	0.295
Normal	85/323 (26.3)	Referent	–	–
1–3 yrs				
Vitamin A levels				
Deficiency	165/229 (72.1)	5.624 (4.132, 7.653)	5.434 (3.962, 7.455)	<0.001
Subclinical deficiencies	431/1,008 (42.8)	1.629 (1.381, 1.922)	1.636 (1.379, 1.942)	<0.001
Normal	480/1,527 (31.4)	Referent	–	–
3–6 yrs				
Vitamin A levels				
Deficiency	219/266 (82.3)	4.870 (3.499, 6.778)	4.647 (3.316, 6.513)	<0.001
Subclinical deficiencies	648/1,075 (60.3)	1.586 (1.355, 1.857)	1.606 (1.365, 1.889)	<0.001
Normal	753/1,540 (48.9)	Referent	–	–
> 6 yrs				
Vitamin A levels				
Deficiency	88/112 (78.6)	7.346 (4.576, 11.793)	6.990 (4.329, 11.286)	<0.001
Subclinical deficiencies	259/563 (46.0)	1.707 (1.372, 2.124)	1.757 (1.402, 2.202)	<0.001
Normal	283/850 (33.3)	Referent	–	–
Residential location				
Urban				
Vitamin A levels				
Deficiency	395/579 (68.2)	3.611 (2.995, 4.355)	4.165 (3.415, 5.079)	<0.001
Subclinical deficiencies	1,166/2,618 (44.5)	1.351 (1.220, 1.496)	1.445 (1.297, 1.609)	<0.001
Normal	1,375/3,688 (37.3)	Referent	–	–
Migrant population				
Vitamin A levels				
Deficiency	41/54 (75.9)	6.550 (3.364, 12.753)	7.407 (3.583, 15.315)	<0.001
Subclinical deficiencies	132/249 (53.0)	2.343 (1.665, 3.297)	2.845 (1.970, 4.108)	<0.001
Normal	104/320 (32.5)	Referent	–	–
Rural				
Vitamin A levels				
Deficiency	77/88 (87.5)	6.311 (3.190, 12.487)	6.707 (3.258, 13.806)	<0.001
Subclinical deficiencies	144/206 (69.9)	2.094 (1.413, 3.105)	2.345 (1.535, 3.584)	<0.001
Normal	122/232 (52.6)	Referent	–	–
BMI				
Underweight				
Vitamin A levels				
Deficiency	36/49 (73.5)	3.130 (1.565, 6.263)	3.204 (1.546, 6.638)	0.002
Subclinical deficiencies	82/173 (47.4)	1.019 (0.676, 1.534)	1.028 (0.673, 1.570)	0.897
Normal	92/196 (46.9)	Referent	–	–
Normal				
Vitamin A levels				
Deficiency	379/551 (68.8)	3.837 (3.164, 4.654)	4.345 (3.548, 5.322)	<0.001
Subclinical deficiencies	1,116/2,485 (44.9)	1.420 (1.278, 1.577)	1.512 (1.356, 1.686)	<0.001
Normal	1,261/3,457 (36.5)	Referent	–	–
Overweight/obesity				
Vitamin A levels				
Deficiency	96/114 (84.2)	6.342 (3.706, 10.852)	7.529 (4.253, 13.330)	<0.001
Subclinical deficiencies	204/314 (65.0)	2.205 (1.637, 2.971)	2.514 (1.830, 3.452)	<0.001
Normal	201/440 (45.7)	Referent	–	–
Food types				
Staple food				
Vitamin A levels				
Deficiency	395/549 (71.9)	4.359 (3.567, 5.327)	4.573 (3.704, 5.645)	<0.001
Subclinical deficiencies	1,031/2,222 (46.4)	1.471 (1.316, 1.645)	1.578 (1.403, 1.776)	<0.001
Normal	1,105/2,983 (37.0)	Referent	–	–
Vegetables				
Vitamin A levels				
Deficiency	11/17 (64.7)	2.888 (1.024, 8.147)	3.165 (1.052, 9.529)	0.040
Subclinical deficiencies	60/110 (54.5)	1.890 (1.174, 3.044)	2.041 (1.216, 3.426)	0.007
Normal	73/188 (38.8)	Referent	–	–
Eggs white or Egg yolks				
Vitamin A levels				
Deficiency	34/53 (64.2)	3.063 (1.672, 5.613)	4.285 (2.236, 8.210)	<0.001
Subclinical deficiencies	110/250 (44.0)	1.345 (0.960, 1.885)	1.587 (1.099, 2.290)	0.014
Normal	118/320 (36.9)	Referent	–	–
Meat				
Vitamin A levels				
Deficiency	32/38 (84.2)	8.205 (3.346, 20.121)	6.375 (2.545, 15.973)	<0.001
Subclinical deficiencies	103/210 (49.0)	1.481 (1.051, 2.087)	1.304 (0.909, 1.870)	0.149
Normal	143/363 (39.4)	Referent	–	–
Fruits				
Vitamin A levels				
Deficiency	40/58 (69.0)	3.019 (1.669, 5.461)	4.994 (2.509, 9.939)	<0.001
Subclinical deficiencies	132/264 (50.0)	1.358 (0.990, 1.864)	1.576 (1.119, 2.221)	0.009
Normal	159/375 (42.4)	Referent	–	–

* *Adjusted model: adjusted for age, sex, residential location, BMI, and food types*.

## Discussion

The present large-scale cross-sectional study in China found a consistent association between vitamin A deficiency and subclinical vitamin A deficiency and increased risk for RRTIs. A stronger association was also observed between vitamin A deficiency and RRTI risk among elder children, rural and migrant children, children with higher BMI, and those consuming primarily meat products, suggesting that vitamin A supplementation for the prevention of RRTIs might be more necessary among these children.

RTIs are series of respiratory system diseases caused by infectious pathogenic microorganisms. RTIs can be classified into upper respiratory tract infections (including rhinitis, pharyngitis, and laryngitis) and lower respiratory tract infections (including tracheitis, bronchitis, and pneumonia) according to the area of onset (13, 14). Several possible complications, such as acute carditis, nephritis, and rheumatism, can occur in some children. Children with weaker immunity are more susceptible to RTIs, which may even progress to RRTIs, a special type of RTI extremely prevalent among children. Delayed diagnosis or inadequate treatment could induce severe complications and seriously impact healthy growth (15). Researchers have long explored the etiology of RTIs and RRTIs, as well as risk factors of RTIs, focusing on vitamin A status in children.

Several randomized trials and double-blind trials have indicated that vitamin A supplementation might boost the immune system of infected patients suffering from chronic vitamin A deficiency (16–18). Ma et al., who conducted a case–control study that compared patients with acute bronchiolitis and those with other respiratory disease/those who were uninfected, confirmed the association between acute bronchitis infection and vitamin A levels independent of other potential risk factors. After considering potential confounders, the same study found that vitamin A levels remained significantly associated with RRTIs (19). Zhang et al. found that low vitamin A levels were significantly associated with the risk for RRTIs in children (5). Similarly, the present study found a negative association between serum vitamin A levels and risk for RRTIs, as well as RTI symptoms, in children and adolescents aged 6 months to 17 years. Therefore, children's nutrition should be closely monitored to avoid vitamin A insufficiency and subclinical vitamin A deficiency and prevent incidences of RRTIs.

Evidence suggests that vitamin A plays a critical role in body growth and immune system development in mammals. Common infectious diseases, such as measles, diarrhea, and respiratory infection, are a result of weakened immune response (20). Immunoglobulin A (IgA), one of the most important indices of the mucosal immune system, had been confirmed to be associated with respiratory infection (21). Previous studies indicated that vitamin A plays a critical role in T-cell differentiation, IgA secretion, and class switching (22–24). Additionally, vitamin A deficiency can impact mucoprotein expression and decrease lymphocyte proliferation following antigen activation, which could certainly decrease the immune response in the patients' airway mucus (25), which consists of mucoproteins and glycoproteins that form a protective barrier against antigens (26). Moreover, researchers have found that vitamin A-deficient mice exhibited significant impairment in virus-specific IgA and CD8+ T-cells in their airway (27). Taken together, all aforementioned findings suggest that vitamin A deficiency can weaken the immune status and increase the risk of infection. Our results suggested that the incidence of symptomatic RRTIs was low among children with normal vitamin A levels. Further, serum vitamin A levels were negatively correlated with both symptomatic RRTIs and symptomatic acute RTIs and weakly, but non-significantly, correlated with asymptomatic RRTIs in children.

Additionally, the present study found an age difference in the association between vitamin A deficiency and risk for RRTIs. Apart from age, children who were obese/overweight also demonstrated a slightly higher risk for RRTIs compared with those with healthy weight, indicating that age and fat accumulation affect the association between vitamin A levels and risk for RRTIs. However, the underlying mechanism warrants further evaluation. Moreover, our findings revealed a stronger association between vitamin A deficiency and risk for RRTIs among rural and migrant children than among urban children, which could have been due to the combined influence of economic status, nutritional status, diversification or dietary preference, and health concerns.

This relatively large-scale cross-sectional study included 8,034 children with sufficient statistical power to detect the association between vitamin A deficiency and RRTIs, especially among subgroup populations. However, several limitations of this study are worth noting. Given the cross-sectional design of this study, a causal relationship between low serum vitamin A levels and RRTIs could not be confirmed. Moreover, we could not discriminate the time order of vitamin A deficiency and RRTIs. However, our findings reconfirmed the hypotheses of previous studies. We also found that vitamin A deficiency might be associated with increased risk for RRTIs among several specific subpopulations, such as older children, those who are obese/overweight, and rural and migrant children. This indicates that not all children might require vitamin A supplementation, indirectly and partially supporting the results of previous studies demonstrating that vitamin A supplementation produces no substantial reduction in the symptoms or morbidity of respiratory infections (28). Only one study showed that vitamin A supplementation could decrease the mortality of young patients with measles and the severity of measly infection but no other respiratory infections (29). Similarly, high dosages of supplementary vitamin A conversely increase the morbidity of lower respiratory tract infections, which is extremely significant in children with a nutritionally adequate diet (30). Studies have also suggested that only those with undernutrition required vitamin A supplementation to prevent acute RTIs (30). Considering that the participants were enrolled from hospitals, our findings might not be applicable to the community. Children and their parents who visit the hospitals may pay more attention to health and disease and have more knowledge about the disease, making them more likely to report the symptoms. Hence, this study may underestimate the actual prevalence of vitamin A deficiency. RTIs are infectious disease and children's illness may not occur independently, thus the logistic regression model might not be best analysis method for such data. Another limitation is that we did not include detailed dietary information. As is widely known, the dietary composition of children may greatly influence serum vitamin levels. Although a question regarding diet had been included in the questionnaire, it did not provide sufficient details on the dietary composition of children. Thus, further well-designed prospective studies are needed to confirm our findings.

Our findings suggested that vitamin A deficiency was significantly associated with the risk for RRTI and RTI symptoms, especially among older children, children who were overweight/obese, and rural and migrant children. Thus, evaluating the nutritional status of vitamin A and outcomes of supplementation in these children might be an efficient approach toward the prevention of RRTI and RTI symptoms in children. Nevertheless, these findings need to be confirmed by further large-scale prospective studies, especially on the community populations.

## Data Availability Statement

The raw data supporting the conclusions of this article will be made available by the authors, without undue reservation.

## Ethics Statement

The studies involving human participants were reviewed and approved by Ethics Committee of Capital Institute of Pediatrics. Written informed consent to participate in this study was provided by the participants' legal guardian/next of kin.

## Author Contributions

XW contributed to the study design, operation, data collection, and manuscript preparation. XL contributed to the study design, operation, data analysis, manuscript preparation, and manuscript revision. CJ contributed to the study design, operation, data collection, and manuscript revision. XB and XQ contributed to the data analysis, manuscript preparation, and statistical analysis. JW, LZ, NL, NJ, WS, HG, BG, YZ, and LW contributed to operation, data collection, and data analysis. All authors contributed to the article and approved the submitted version.

## Funding

This study was supported by the Clinical Research Project of Development Center for Medical Science and Technology National Health Commission of the People's Republic of China (Grant Number: W2015EAE001).

## Conflict of Interest

The authors declare that the research was conducted in the absence of any commercial or financial relationships that could be construed as a potential conflict of interest. The reviewer KY declared a shared affiliation, with several of the authors XL, CJ, and XB to the handling editor at the time of the review.

## Publisher's Note

All claims expressed in this article are solely those of the authors and do not necessarily represent those of their affiliated organizations, or those of the publisher, the editors and the reviewers. Any product that may be evaluated in this article, or claim that may be made by its manufacturer, is not guaranteed or endorsed by the publisher.
